# Hierarchical Tactile-Based Control Decomposition of Dexterous In-Hand Manipulation Tasks

**DOI:** 10.3389/frobt.2020.521448

**Published:** 2020-11-19

**Authors:** Filipe Veiga, Riad Akrour, Jan Peters

**Affiliations:** ^1^Computer Science and Artificial Intelligence Laboratory (CSAIL), Massachusetts Institute of Technology, Cambridge, MA, United States; ^2^Intelligent Autonomous Systems, Technische Universität Darmstadt, Darmstadt, Germany; ^3^Max-Planck-Institut für Intelligente Systeme, Tübingen, Germany

**Keywords:** tactile sensation and sensors, robotics, in-hand manipulation, hierarchical control, reinforcement learning

## Abstract

In-hand manipulation and grasp adjustment with dexterous robotic hands is a complex problem that not only requires highly coordinated finger movements but also deals with interaction variability. The control problem becomes even more complex when introducing tactile information into the feedback loop. Traditional approaches do not consider tactile feedback and attempt to solve the problem either by relying on complex models that are not always readily available or by constraining the problem in order to make it more tractable. In this paper, we propose a hierarchical control approach where a higher level policy is learned through reinforcement learning, while low level controllers ensure grip stability throughout the manipulation action. The low level controllers are independent grip stabilization controllers based on tactile feedback. The independent controllers allow reinforcement learning approaches to explore the manipulation tasks state-action space in a more structured manner. We show that this structure allows learning the unconstrained task with RL methods that cannot learn it in a non-hierarchical setting. The low level controllers also provide an abstraction to the tactile sensors input, allowing transfer to real robot platforms. We show preliminary results of the transfer of policies trained in simulation to the real robot hand.

## 1. Introduction

Dexterous in-hand manipulation is a long studied problem, involving precise movement, inter-finger coordination, and contact management (Okamura et al., 2000). While manipulating objects within a grip is possible with simple grippers, external forces such as gravity or interactions with the environment are necessary to generate the manipulation movements (Dafle et al., 2014; Chavan-Dafle and Rodriguez, 2015; Stork et al., 2015). When considering dexterous hands, the problem complexity greatly increases (Ma and Dollar, 2011), as the additional fingers allow for an increased number of possible solutions for each manipulation action and a larger number of possible interactions with objects. Traditional in-hand manipulation control approaches tackle simplifications of the general problem by attempting small movements or by relying on several strong assumptions regarding contact and the precision of the available robot and object models (Maekawa et al., 1995; Zheng et al., 2000; Bai and Liu, 2014). Even with such simplifications, experiments on real robot platforms are prohibitively hard and thus frequently omitted in the literature (Zheng et al., 2000; Bai and Liu, 2014). Seeing in-hand manipulation as a planning or optimization problem provides solutions for more general forms of the problem but most of these solutions integrate very little to no feedback (Cherif and Gupta, 1999; Saut et al., 2007; Mordatch et al., 2012; Sundaralingam and Hermans, 2018). Considering feedback during task execution is crucial to tackle the variability introduced by objects, in the form of distinct shapes, surface properties, target movements, or initial grasp configurations. To achieve a sufficiently general solution to in-hand manipulation, manipulation controllers not only have to generate suitable trajectories that take into account task variability but also have to adapt in accordance with the feedback signals observed during task execution to compensate for unforeseen events, such as object slip.

Tactile sensing is an attractive form of feedback for in-hand manipulation, as it provides information directly from the interaction points. It offers substantial advantages over other forms of feedback such as vision and force, by disregarding effects such as occlusion while providing rich information at high frequencies (Yousef et al., 2011). Additionally, tactile information has been shown to help with the interaction variability required for in-hand manipulation, as it enabled objects to be grasped robustly regardless of their shape or material properties (Veiga et al., 2020). However, integrating high dimensional tactile feedback signals in the control loop of an already complex in-hand manipulation controller is non-trivial.

Reinforcement Learning (RL) has found great success in solving control tasks with large input spaces on both simulated (Mnih et al., 2015; Silver et al., 2016) and physical platforms (Levine et al., 2016). Thus, several approaches based on reinforcement learning (Van Hoof et al., 2015; Popov et al., 2017; Akkaya et al., 2019; Zhu et al., 2019; Andrychowicz et al., 2020), learning from demonstration (LfD) (Li et al., 2014), combinations of RL and LfD (Gupta et al., 2016; Rajeswaran et al., 2017), or optimal control with learned local models (Kumar et al., 2016) have been proposed for in-hand manipulation. Despite this, when considering complex tactile sensors such as the BioTac (Wettels et al., 2014), the richness of the feedback signals leads to considerably more complex state spaces and transition functions, yielding significantly more challenging RL problems. For example, pressure on different contact points of the BioTac sensors is measured from the displacement of fluid within the fingertip which results from the deformation of its malleable skin. Such a complex physical process is currently impossible to simulate accurately and efficiently. Hence any RL policy learned in simulation using a model of the tactile sensor would not transfer to a physical robot. On the other hand, learning the task directly on the robot is hardly feasible because (i) in-hand manipulation tasks are contact-rich, which creates non-linearities in the state transitions and precludes the learning of a forward dynamics model in a model-based RL setting and (ii) the high dimensionality of the tactile sensors precludes the use of model-free RL directly on the robot due to a prohibitively high sample complexity. Accordingly, only Van Hoof et al. (2015) use RL with integrated tactile information by training the policy directly on a real robot and using very simple tactile information. Additionally, the task is constrained during training by having the object supported by an external surface that prevents it from falling.

**Figure 1:**
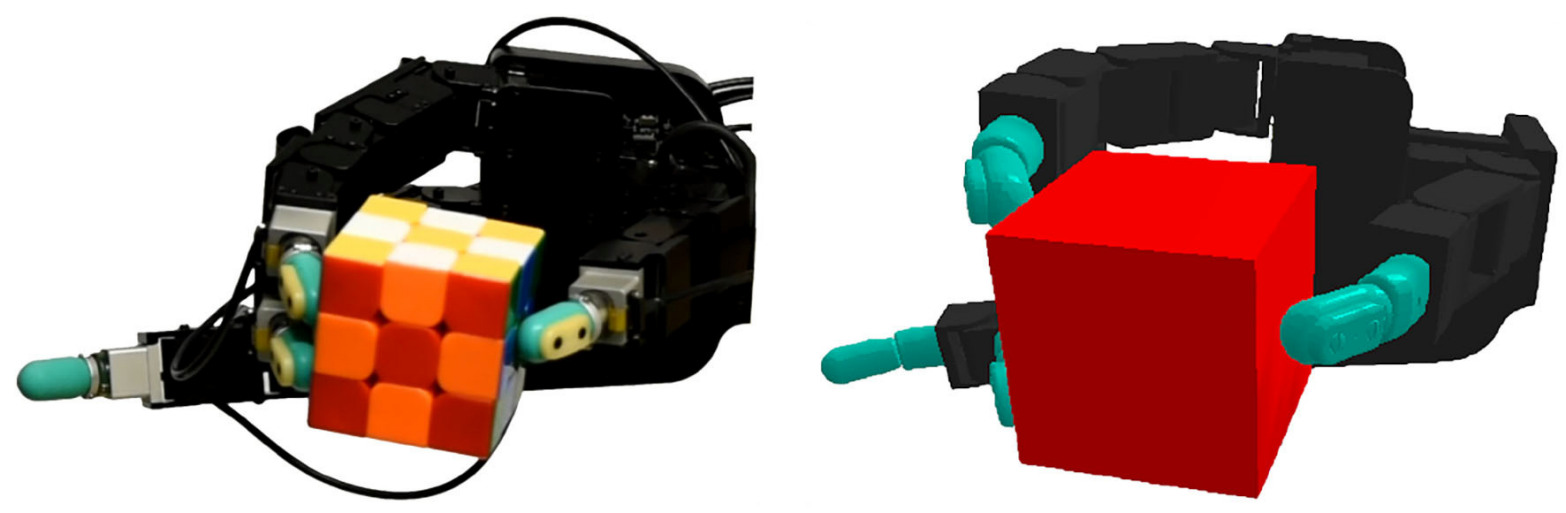
The real **(left)** and simulated **(right)** Allegro hands that were used in our experiments. The tactile information provided by the sensors on the real platform is abstracted in simulation by using our proposed hierarchical control decomposition.

Constraining the manipulation task to a position where the object is in a supported position (either by the palm of the robot or by an external support surface), such that the object is less likely to be dropped during exploration, is common among several proposed approaches (Van Hoof et al., 2015; Kumar et al., 2016; Rajeswaran et al., 2017; Akkaya et al., 2019; Andrychowicz et al., 2020). The use of such a constrain is justified by the complex nature of the transition function of in-hand manipulation tasks, even in simulated environments. Indeed, if the robot is holding an object as in Figure 1, any exploratory action (a random perturbation to the current joint position) is likely to make the object fall and thus terminates the trajectory after only a few number of steps. We observed that such exploratory behavior could lead to premature convergence of RL to poor local optima where the robot reinforces behaviors that throw the object toward the target. This results in a short term accumulation of rewards at the detriment of the longer term rewards. In addition, we observed that methods such as the ones used in Andrychowicz et al. (2020) to produce impressive results on a real robot with the object supported by the palm, are unable to learn the task when the support is removed.

To address both the learning of in-hand manipulation tasks with rich tactile feedback and the relaxing of the support constraint, we propose a hierarchical control decomposition that relies on a low-level control scheme, composed by a set of independent stabilization controllers, to keep the object firmly gripped during the manipulation actions. These low level stabilization controllers have the benefits of (i) enabling the efficient learning of complex in-hand movements in simulation by maintaining the object in the robot's hand for a longer period of time, simplifying the transition function and (ii) potentially allowing transfer of policies learned in simulation to physical environments by abstracting tactile information and letting the upper level policy be solely defined on joint information. The stabilization controllers are highly inspired by neurophysiological studies (Johansson, 1996; Flanagan et al., 2006) and have been extensively studied in prior work (Veiga et al., 2020). We show that with the proposed hierarchical decomposition RL methods are able to learn complex and generalizable manipulation actions.

## 2. Hierarchical Control Decomposition for In-Hand Manipulation

In order to learn general manipulation policies in simulation, that can transfer to a physical robot, we propose a hierarchical control decomposition composed of two control levels: a set of grip stabilization controllers running independently on each finger and a manipulation movement policy that produces the movement trajectory in joint space and trades-off between manipulation and stabilization. We begin by defining the RL problem in a non-hierarchical fashion, followed by a description of the stabilizers that compose the low-level of our proposed hierarchical decomposition and showcase the differences between the non-hierarchical and the hierarchical learning problems.

### 2.1. Reinforcement Learning Problem Definition

**Figure 2:**
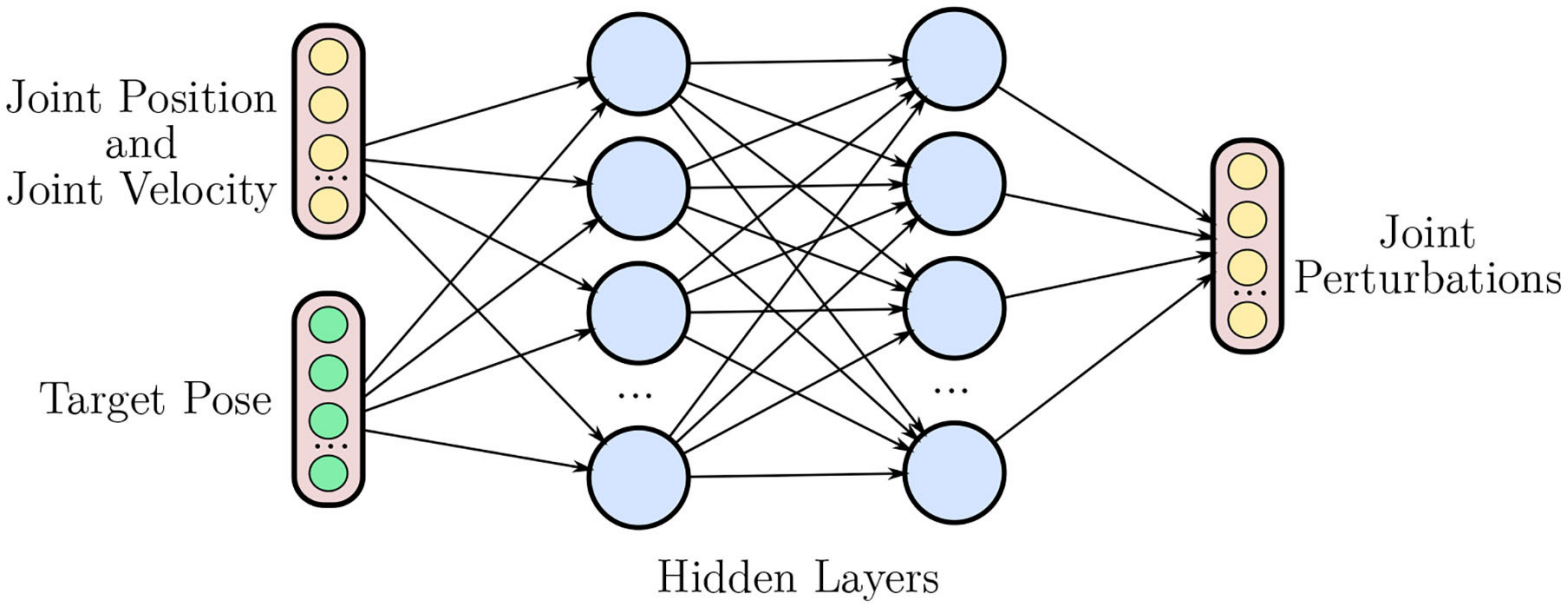
Overview of an non-hierarchical policy network. The network takes joint positions *q*, joint velocities $\dot{q}$, and the target pose *T* as inputs, outputting a set of perturbations to the current joint position *u*_*mov*_.

Given an initial grasp on an object, we consider the in-hand manipulation task of translating and/or rotating the object to a target pose. We phrase this problem as a Markov Decision Process (MDP), defined by the quintuple (***S**, **A**, R, P*, γ), where ***S*** represents the state space, ***A*** the action space, *P*(*s*_*t*+1_|*s*_*t*_, *a*_*t*_) the transition probability, *R*(*s*_*t*_, *a*_*t*_) its associated reward, and γ is the discount factor. In a non-hierarchical RL setting (NH-RL), the state space is comprised of joint positions *q*, joint velocities $\dot{q}$ and target pose *T*. The action space is the set of perturbations to the current joint position *u*_mov_, constrained by a maximum tolerated velocity. The structure of the non-hierarchical neural network policies is depicted in Figure 2. The reward *R*(*s*_*t*_, *a*_*t*_) is inversely proportional to the distance between the current and target object coordinates.

Let π be a stochastic policy giving the probability π(*a*|*s*) of executing action *a* ∈ ***A*** in state *s* ∈ ***S***. Let the Q-function be $Q_{\pi }\left(s,a\right)=\text{E}\left[\overset{\infty }{\underset{t=0}{\sum }}\gamma^{t}R\left(s_{t},a_{t}\right)|s_{0}=s,a_{0}=a\right]$, where the expectation is taken w.r.t. all random variables *s*_*t*_ and *a*_*t*_ for *t* > 0. Let *V*_π_(*s*) = **E**_*a*~π_[*Q*_π_(*s, a*)]. The goal of RL is to find the policy maximizing the policy return *J*(π) = *V*_π_(*s*_0_) where *s*_0_ denotes some initial state (an initial grasp in our case).

### 2.2. Independent Grip Stabilization Control

The stabilization controllers that compose the base control level where introduced in Veiga et al. (2020), and are deployed on each finger independently. By interpreting the tactile signals provided by the BioTac sensors (Wettels et al., 2014), these independent finger stabilizers (IFS) locally avoid predicted slip events. This allows them to keep objects stable within multi fingered grips while not being constrained to a particular grasp configuration or a particular distribution of force between the fingers. In a hierarchical setting, the main task of the stabilizers is to ensure grip stability throughout the manipulation action. Formally, provided with a label *c*_*t*+τ_*f*__ ∈ [slip, ¬contact, contact] from a learned tactile based slip predictor, where τ_*f*_ is the prediction window of the predictors, the level of a leaky integrator at time *t*, denoted *y*_*t*_, is adjusted as follows

(1) $$\begin{array}{r} y_{t}=\beta y_{t-1}+\left(1-\beta \right)L \end{array}$$

where β is the leakage at each time step and

(2) $$\begin{array}{l} L=\{\begin{array}{ll} 1 & \text{if }c_{t+\tau_{f}}=\text{slip},\\ 0 & \text{otherwise} \end{array} \end{array}$$

is the integrator input. The integrator value is then used by the stabilizer to regulate the desired task-space velocity in the contact normal direction, i.e.,

(3) $$\begin{array}{l} {\text{v}}_{\text{stab}}={\text{N}}_{t}y_{t}, \end{array}$$

where **N**_*t*_ is a unit vector pointing in the contact normal direction. In short, the integrator changes with the predicted contact state, accumulating its response when slip is predicted and leaking if contact. Finally, the stabilization disturbances to the joint positions of the i-th finger ${\text{u}}_{\text{stab}}^{i}$ are calculated using inverse kinematics.

There are three differences in implementation pertaining to these controllers between the simulated and real robot environments. The first, is the manner in which the normal contact direction is acquired. In simulation, the contact normal is acquired via the simulator's collision engine while the real robot estimates it via the weighted average of the normal directions of the electrodes. The weights are the activations of each electrode as described in Wettels et al. (2009). The second difference concerns the intensity of the stabilizer response. Due to fluctuations of the fluid of the real sensors, pressure values might indicate that there is no longer contact for one time step, creating jerky responses. As in Veiga et al. (2020), the controllers of the real robot do not immediately stop whenever contact is lost, but have their response smoothly reduced over a period of 200 ms. The final difference concerns the slip signals used by the stabilizers. In simulation, slip signals are provided by a heuristic based slip detector, that observes the changes in relative position and orientation between the fingertip and the object to detect slip. In the real robot, slip is predicted from learned tactile based slip predictors, as described in Veiga et al. (2020), and a prediction window τ_*f*_ of ten is used.

The stabilization controllers are independent of the nature of the manipulation task (e.g., nature of the manipulated object, target coordinates, or type of initial grasp) and do not need to be learned. Most importantly, they provide an abstraction to the tactile information provided by the sensors, allowing the high level movement policy to not depend on tactile information while the overall system still reacts to tactile feedback. Being able to learn movement policies with information that is readily available to both the simulated and the real robot facilitates the transfer of policies between the two.

### 2.3. In-Hand Manipulation Movement Policy

To generate the manipulation movements, a high-level policy π_θ_, parameterized by the weights of a neural network θ, is learned in a simulation environment depicted in Figure 1. In contrast to the NH-RL case, in the hierarchical RL (H-RL) setting, the new state space ***S***′ is not only comprised of joint positions *q*, joint velocities $\dot{q}$ and target pose *T* but also includes the state **y** = [*y*^1^, …, *y*^4^] and the state variations $\Delta \text{y}=\left[y_{t}^{1}-y_{t-1}^{1},...,y_{t}^{4}-y_{t-1}^{4}\right]$ of all the finger stabilizers. The action space is also different, with the new action space $A^{\prime }=A\times {\left[0,1\right]}^{N_{\text{fing}}}$, now including a set of *N*_fing_ uni-dimensional merging coefficients α_*i*_, where *N*_fing_ is the total number of fingers, in addition to the movement commands in the form of perturbations to the hand's joint positions **u**_mov_, that were already included in the action space ***A***. The merging coefficients α_*i*_ regulate the combination of both perturbations, ${\text{u}}_{\text{stab}}^{i}$ and **u**_mov_, to compose the final action. Letting ${\text{u}}_{i}^{*}$ be the combined response of each individual finger

(4) $$\begin{array}{l} {\text{u}}_{i}^{*}=\alpha_{i}{\text{u}}_{\text{mov}}^{i}+\left(1-\alpha_{i}\right){\text{u}}_{\text{stab}}^{i}. \end{array}$$

**Figure 3:**
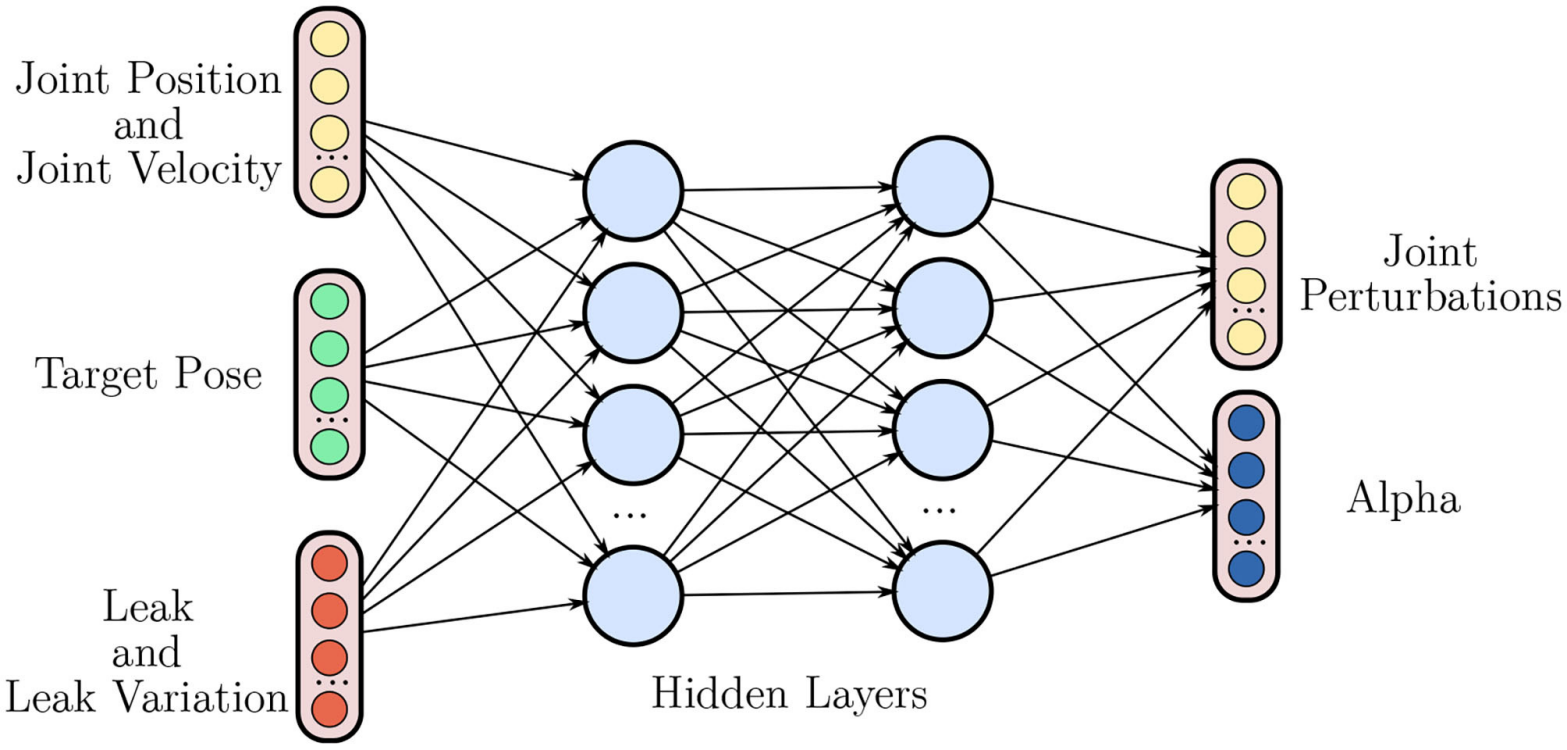
Overview of the high level policy network that produces the manipulation movements. As in the NH-RL case, the network takes joint positions *q*, joint velocities $\dot{q}$, and the target pose *T* as inputs but now also receives the state of the stabilizers *y* and its variations Δ*y*. The movements are once again represented by a set of perturbations to the current joint position *u*_*mov*_. The network now also outputs the merging coefficients α_*i*_ between the movement commands **u**_mov_ and each of the stabilizers responses ${\text{u}}_{\text{stab}}^{i}$.

**Figure 4:**
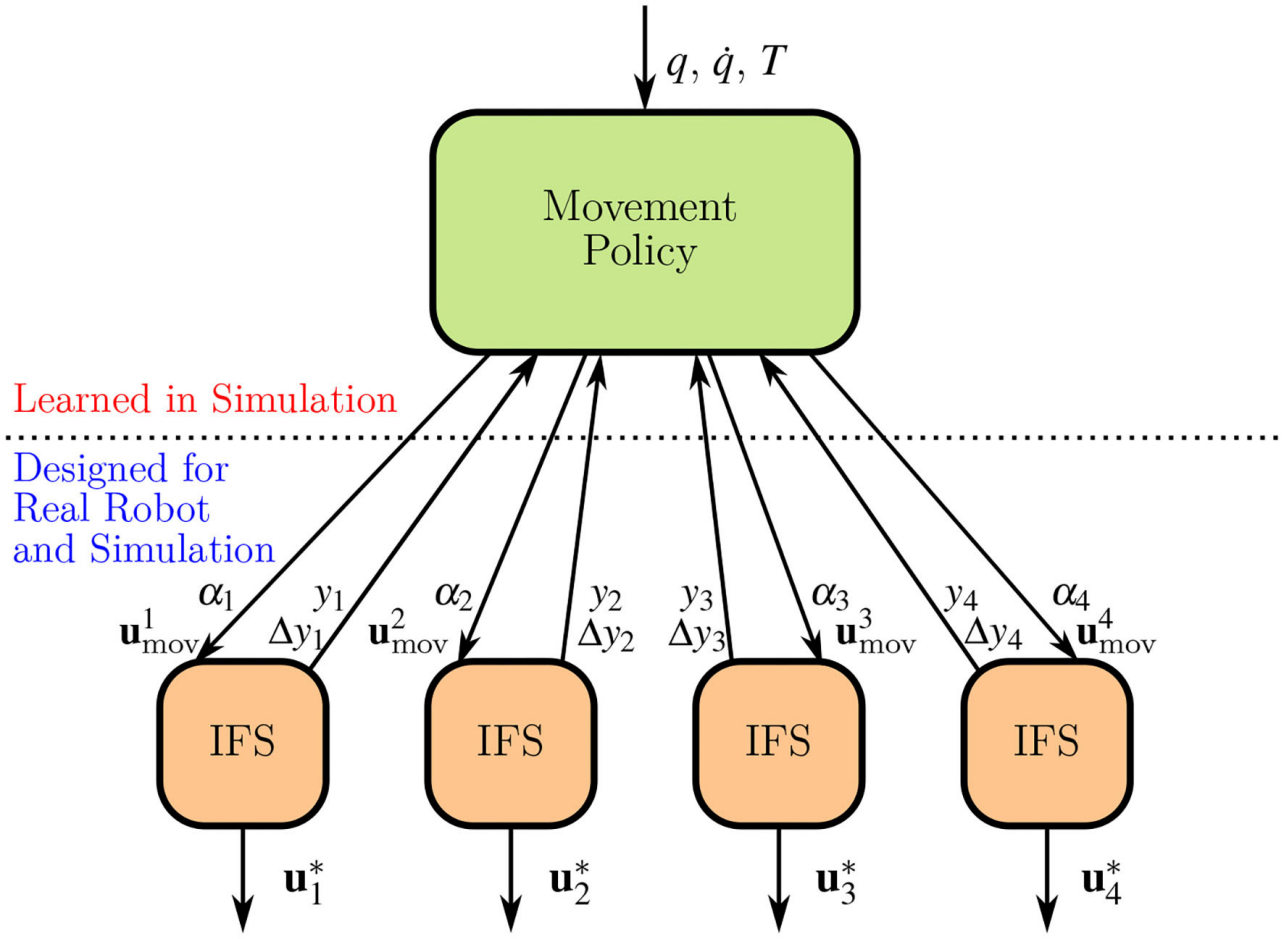
The proposed hierarchical structure. The movement policy has access to joint positions *q*, joint velocities $\dot{q}$ target pose *T*, and the internal state variables of each of the individual stabilizers *y*_*i*_ and Δ*y*_*i*_. To each stabilizer, it transmits a set of joint perturbations ${\text{u}}_{\text{mov}}^{i}$, that generate the necessary finger movements, in addition to a coefficient α_*i*_ used to merge movement and stabilizing perturbations ${\text{u}}_{\text{stab}}^{i}$, and generate the final command $u_{i}^{*}$.

Figure 3 depicts the high-level movement policy of the H-RL setting while Figure 4 provides an overview of the proposed hierarchy. The latter also re-emphasizes the fact that low-level is designed both in simulation and on the real robot, allowing the high-level policy to rely solely on joint space information.

An important set of hyper-parameters in our hierarchical decomposition is the initial distribution of each α_*i*_, in order to obtain maximum variability in the trajectories of the initial policy and facilitate the RL process. Low values of α_*i*_ have the desired effect of stabilizing the grip but dampen the variability of the initial trajectories. Similarly, high values of α_*i*_ produce trajectories with low variability as the object falls almost immediately. To find an appropriate trade-off we manually tune the hyper-parameters governing the distribution of α by visual inspection of the resulting initial policy in simulation. The resulting distribution for each α_*i*_ is a Gaussian with mean 0.5 and a variance of 0.25. By centering the distribution at the transition point between the stabilization and the movement perturbations, we allow for exploration movements with stabilizer compensation. The variance being relatively low prevents sudden shifts from full movement to full stabilization perturbation and vice-versa.

Any RL algorithm can be applied to this hierarchical decomposition as the actions are not time-extended. Learning proceeds as follow: at the start of an episode a random target coordinate is sampled and the policy is executed until the object falls or 3,000 time steps (10 s) have elapsed. Upon collection of the trajectories we use TRPO (Schulman et al., 2015) to update the neural network policy depicted in Figure 3. In our experiments, the same implementation of TRPO (Dhariwal et al., 2017) is used to compare both NH-RL, and the proposed H-RL to in-hand manipulation.

## 3. Experimental Evaluation

Using a simulated environment, we evaluate the efficiency of our proposed H-RL when compared to NH-RL and present preliminary results on transferring H-RL policies learned in simulation to a real robot platform.

### 3.1. Experimental Procedure, Testing Platform, and Tactile Sensors

All experiments are performed either on a simulated or real version of the Allegro Hand that is equipped with BioTac fingertip sensors (SynTouch Inc., www.syntouchinc.com). The Allegro Hand (Wonik Robotics GmbH, www.simlab.co.kr), is a four fingered hand with four joints per finger, for a total of 16 actuated degrees of freedom. With the exception of the thumb, all fingers have two metacarpal joints (rotation and flexing), a proximal joint and a distal joint. The thumb does not have a distal joint having an abduction joint instead. A PD controller was used to control the robot joint positions with a control loop that runs at 300 Hz.

BioTac tactile sensors (Wettels et al., 2014) were used as fingertip sensors. The sensors provide multi-modal responses composed of low and high frequency pressure (*P*_dc_ and *P*_ac_) captured by a pressure transducer, local skin deformations (*E*) acquired through local impedance changes measured by 19 electrodes scattered across the sensors core surface, as well as temperature and thermal flow (*T*_dc_ and *T*_ac_) measured by a thermometer. All data channels of the sensor are sampled at a rate of 100 Hz. The high frequency pressure is sampled in batches of 22 values at the same frequency. Considering all channels and the *P*_ac_ batch data, the sensors outputs a total of 44 values every 10 ms.

The PyBullet simulation environment (Coumans and Bai, 2018) is used to simulate the hand and the fingertip sensors. The PD control gains of the hand were tuned in simulation to emulate the behavior of the real hand. The BioTacs are not simulated. Instead information of contact force and normal direction is obtained directly from the collision engine. In addition to the simulated slip stabilizers, a simplified version of the stabilizers, that uses a constant desired velocity factor β, was implemented and compared with the full stabilizers

(5) $$\begin{array}{l} {\text{u}}_{\text{stab}}^{i}=\beta {\text{N}}_{i}. \end{array}$$

After initial testing, it was found that the simple stabilizers would either apply forces that are not sufficiently strong to keep the object in hand or would apply excess force, hindering the manipulation movements. This is due to the simple stabilizers inability to regulate the applied velocity, and suggests that the lower level of the hierarchy requires feedback in order to be beneficial to the systems performance. These observations led us to present all results using the full stabilizers.

**Figure 5:**
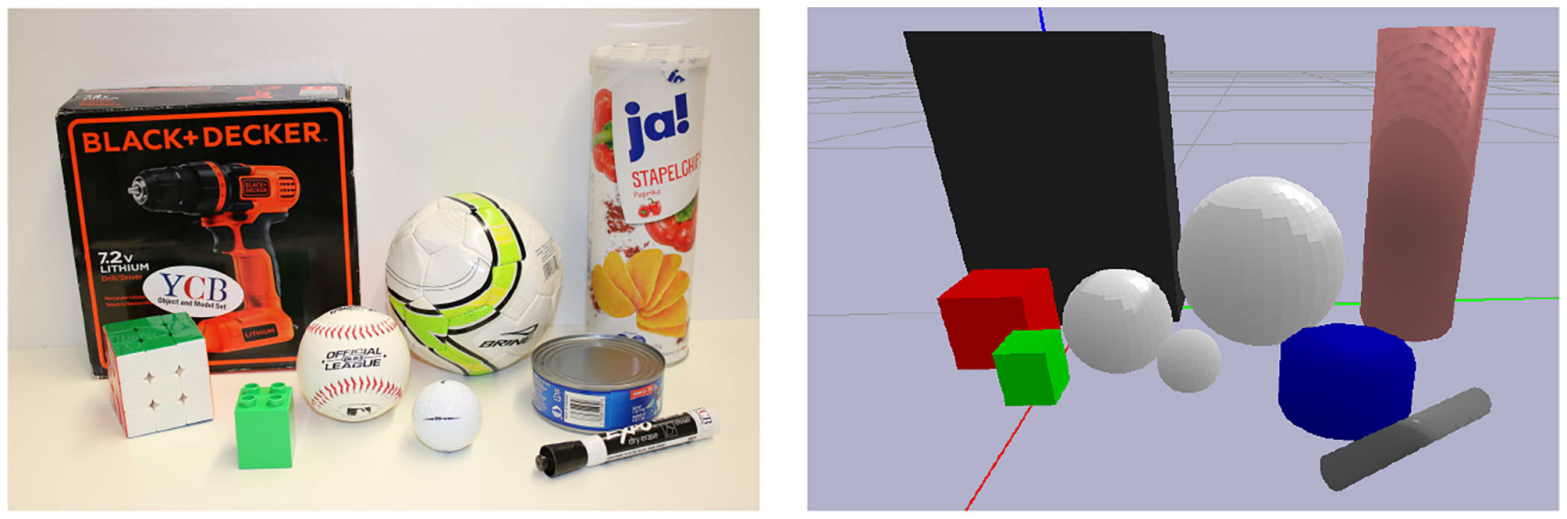
The real **(left)** and simulated **(right)** objects used in our experiments. The objects are a subset of the YCB object and model data set (Calli et al., 2015). Since the stabilizers implemented here have a fixed response along the normal direction, the chips can of the data set was replaced by a similar but empty chips can in order to avoid manipulating heavier objects.

All experiments are performed on a subset of objects from the YCB object and model set (Calli et al., 2015), either simulated or on the real robot, as shown in Figure 5.

**Figure 6:**
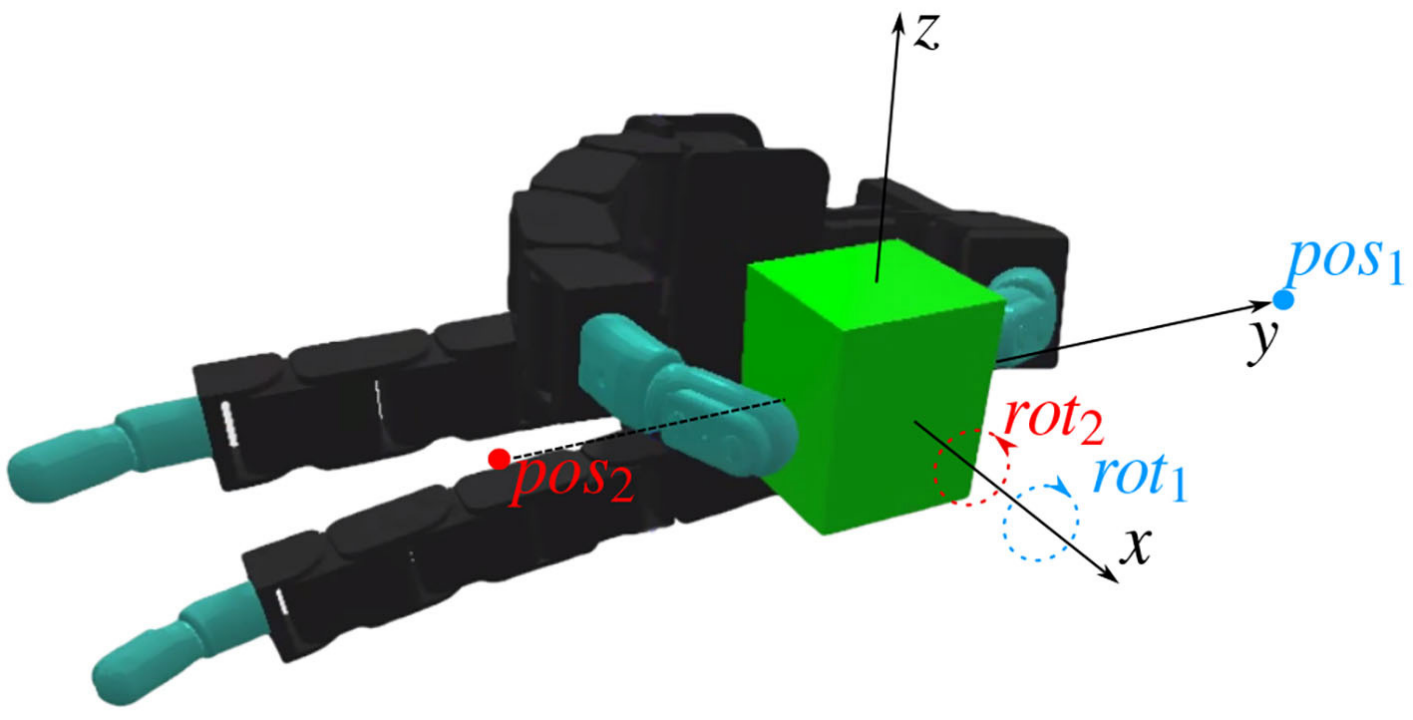
A lego block in the initial grasp position. The coordinate frames defined for the manipulation are shown, as well as the two position targets *pos*_1_ and *pos*_2_, and the two rotation targets *rot*_1_ and *rot*_2_. The position targets represent object translations of 2 cm along the *y* axis. The rotation targets represent clockwise or counter-clockwise rotations of π/4 radians with respect to the *x* axis.

The simulation experiments considered three possible initial configurations: two fingered grasps for the green Lego brick, the golf ball and the marker, three fingered grasps for the Rubik's cube, the baseball and the tuna can and finally four fingered grasps for the screw-driver box, the small football, and the chips can. Each of these state configurations served as the initial pose for four different manipulation movements. These movements were sampled at the beginning of each trial by setting different target positions and target orientations, both with respect to the initial object position. The position targets are sampled from a set of two positions, attempting to move the object by 2 cm to the edge of the work space with respect to the y axis. The hand is oriented such that x is the axis moving away from the palm, y the axis pointing from the palms to the fingers when the fingers are in a stretched position, and z is the height. Rotation targets are either positive or negative π/4 rotations around the initial position with the sign sampled uniformly at random. The coordinate frames, position targets, and rotation targets are depicted in Figure 6. Having four combinations at the edge of the work space allows all target poses to be consistently observed every episode, simplifying the learning process while potentially allowing the policy to generalize to other intermediate poses. Five learning trials were performed for each combination of manipulation/object configuration and target movements with 50 million samples per trial.

Regarding the learning process, the reward function *R* is given by

(6) $$\begin{array}{l} R\left(s_{t},a_{t}\right)=F\left(P_{err}+O_{err}\right)-{\text{a}}_{cost}-{\dot{\text{a}}}_{cost}-d \end{array}$$

where *P*_*err*_ and *O*_*err*_ respectively correspond to the position and orientation terms

(7) $$\begin{array}{l} P_{err}=e^{-{\left({\text{p}}_{curr}-{\text{p}}_{des}\right)}^{2}} \end{array}$$

(8) $$\begin{array}{l} O_{err}=e^{-{\left({\text{o}}_{curr}-{\text{o}}_{des}\right)}^{2}}. \end{array}$$

Using an exponential form for these terms guarantees that the maximum instantaneous reward for each term is one when the error is zero. In order to produce structured manipulation movements, we enforce that the number of fingers in the initial grasp is maintained throughout the manipulation action. This is achieved via *F*, a ratio between the number of fingers initially in contact with the object *K* and the current fingers in contact with the object

(9) $$\begin{array}{l} F=\frac{1}{K}\overset{K}{\underset{k=0}{\sum }}f_{k} \end{array}$$

where *f*_*k*_ equals one if k-th finger is in contact and is zero otherwise. Since both *P*_*err*_ and *O*_*err*_ respectively increase as the position and rotation errors decrease, multiplying the sum of these terms by the ratio *F* effectively limits the instantaneous reward, only providing a fraction of it if the number of fingers is not maintained throughout the trajectory. We also wish to enforce smooth movement during the manipulation action. We do so by applying costs **a**_*cost*_ and ${\dot{\text{a}}}_{cost}$ on the velocity and acceleration respectively

(10) $$\begin{array}{l} {\text{a}}_{cost}=\overset{J}{\underset{i=0}{\sum }}{\left(a_{t}^{i}\right)}^{2} \end{array}$$

(11) $$\begin{array}{l} {\dot{\text{a}}}_{cost}=\overset{J}{\underset{i=0}{\sum }}{\left(a_{t}^{i}-a_{t-1}^{i}\right)}^{2} \end{array}$$

where *J* is the number of joints. Finally, *d* is a negative penalty given when the object is dropped.

In addition to the previous reward terms, a specific term is added to the reward calculation in the H-RL setting. This term is an additional cost

(12) $$\begin{array}{l} {\dot{\alpha }}_{cost}=\overset{N_{\text{fing}}}{\underset{i=0}{\sum }}{\left(\alpha_{t}^{i}-\alpha_{t-1}^{i}\right)}^{2} \end{array}$$

applied on the variation of the α_*i*_. It serves to penalize policies that shift very abruptly between stabilization and movement commands.

### 3.2. Hierarchical-RL vs. Non Hierarchical-RL

**Figure 7:**
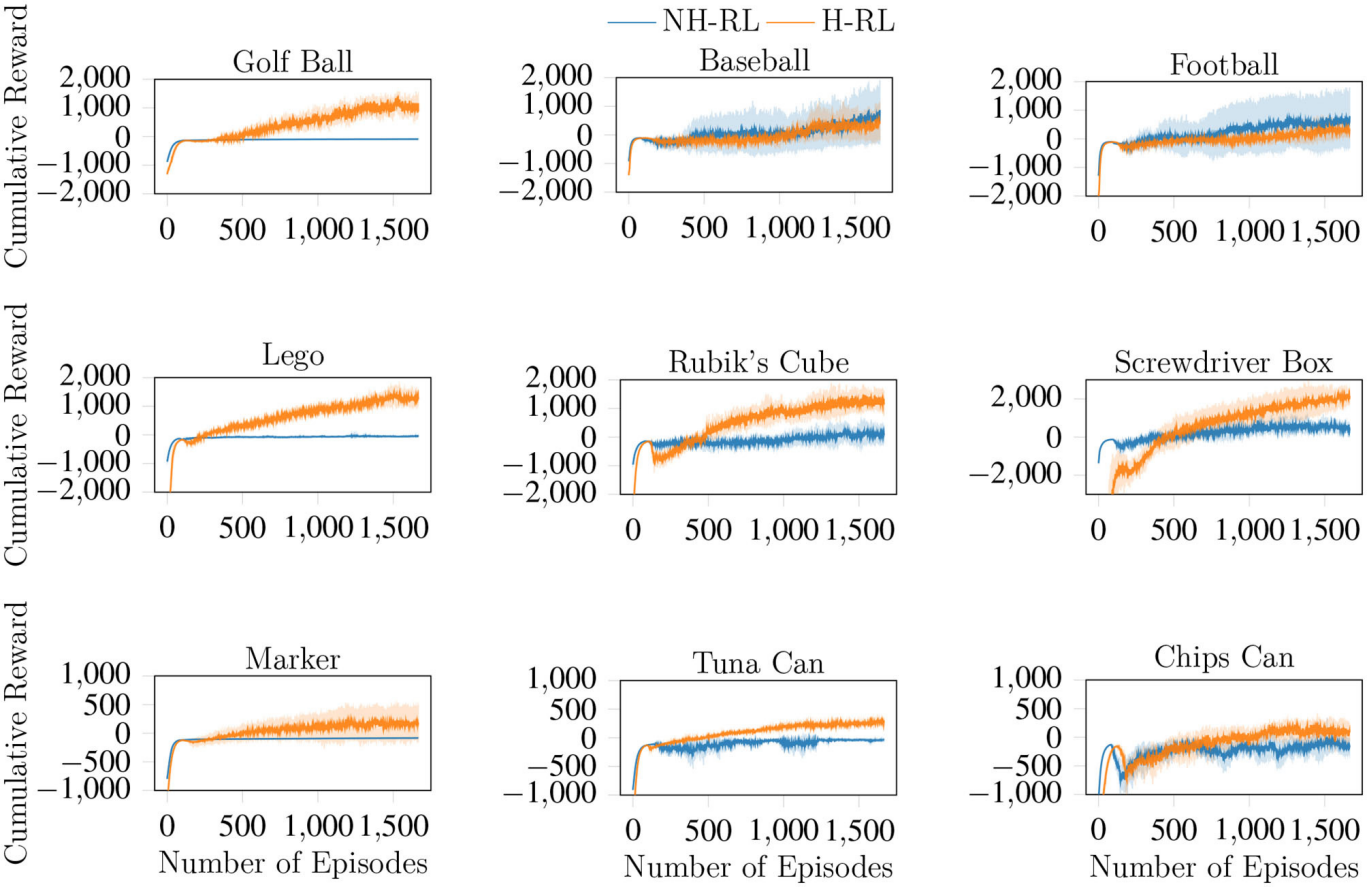
Cumulative reward curves for H-RL and NH-RL, both trained with TRPO. H-RL outperforms NH-RL for smaller objects but the gap in accumulated rewards significantly decreases with object size, with NH-RL showing similar or slightly higher accumulated reward values for larger objects.

**Figure 8:**
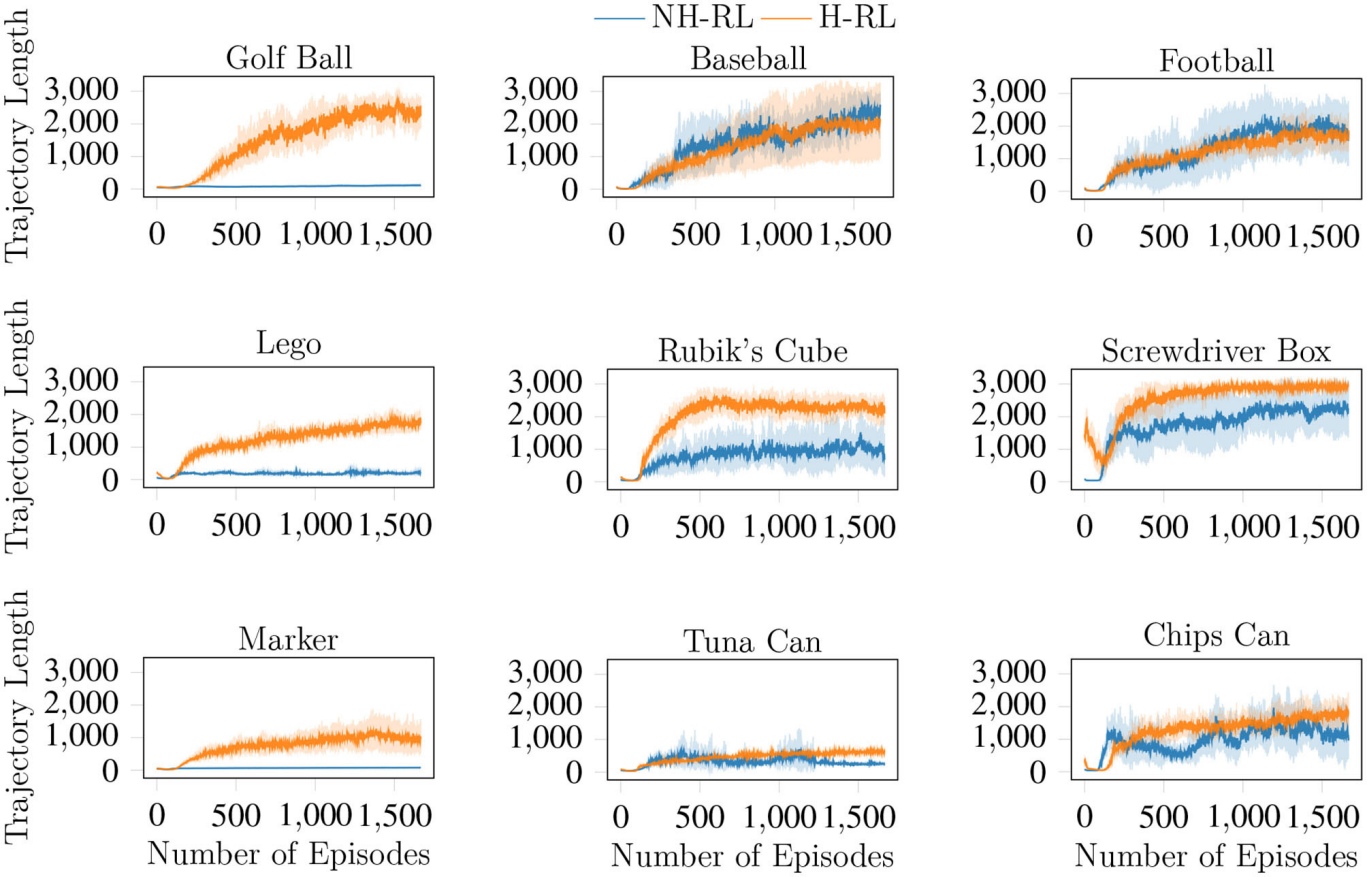
Evolution of the average trajectory lengths achieved (in terms of the number of time steps) by both NH-RL and H-RL with the number of learning episodes. The critical effect of the RL's exploratory actions is evident for smaller objects, where NH-RL is unable to increase the trajectory length, and hence unable to learn.

**Figure 9:**
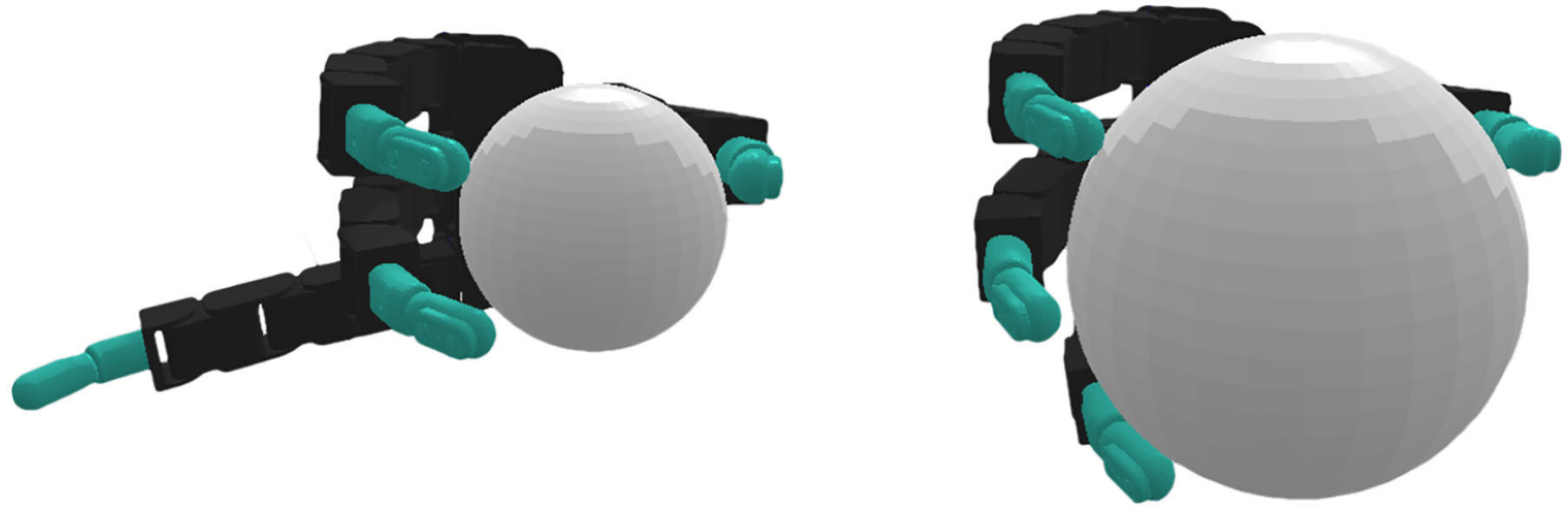
Initial grasps for **(left)** the baseball and **(right)** the mini football. In both cases, one of the fingers is slightly under one of the spheres, acting as a supporting surface and minimizing the effects of the exploratory actions.

We compare the average accumulated reward (cumulative reward) achieved by NH-RL and by our proposed hierarchical decomposition H-RL, respectively represented by the blue and orange curves in Figure 7. Results show that H-RL performs better or on par with NH-RL for all objects. For larger objects such as the football, the screwdriver box, and the chips can, exploratory actions that cause the object to shift in-hand are not as detrimental to the learning episode, as the size of the object allows it to be re-grasped before being dropped. This behavior is shown by a clear correlation between the difference in performance of the two approaches and the size of the object and/or the number of fingers involved in the manipulation action. The impact of bad exploratory actions on the learning process increases as the objects size decreases, rendering NH-RL unable to learn movement policies for smaller objects, while H-RL can learn movement policies for all objects. Moreover, these results are additionally emphasized by the evolution of the trajectory length during learning, shown in Figure 8. The average trajectory length for NH-RL policies remains very close to zero in all experiments with smaller objects, where exploratory actions have a critical effect on the movement. In addition to size, the initial grasp configurations can also greatly influence the outcome of the learning. This is the case for the football and the baseball, where one of the fingers is slightly underneath the object as depicted in Figure 9, serving as support for the exploration actions.

**Figure 10:**
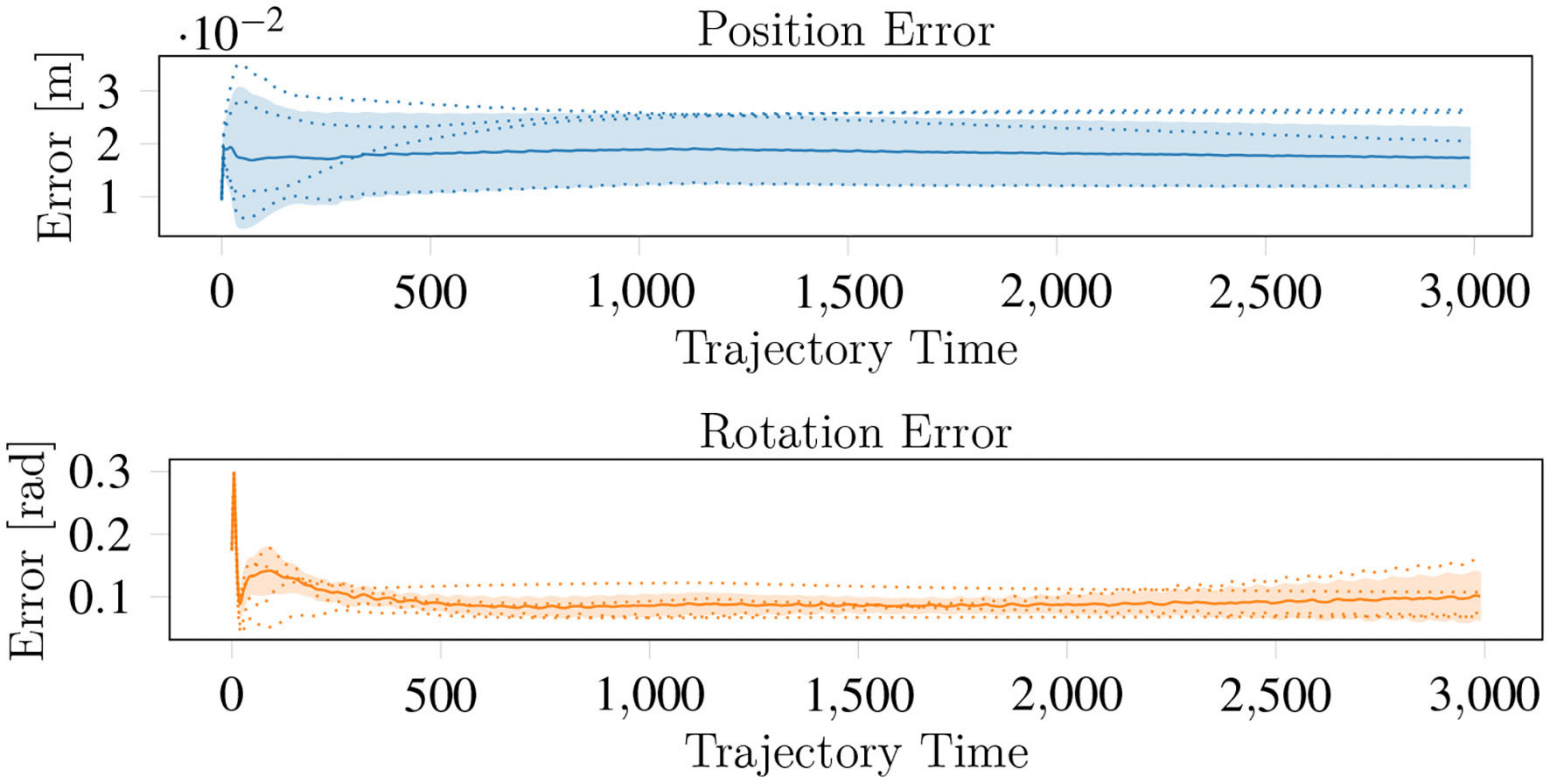
The average behavior of the H-RL movement policies for the lego. The solid lines represent mean and the dashed lines the individual trials. While the position error is maintained through the movement execution, the orientation error is consistently reduced.

The effectiveness of the policies learned by both approaches also substantially differs. While NH-RL is capable of learning policies for the partially supported and for the larger objects, the resulting policies are only capable of maintaining the objects in-hand without any consistent movement toward the target pose. In contrast, the H-RL policies are capable of consistently reorienting the objects to the correct orientations, despite maintaining the initial position error. This behavior, shown in Figure 10 for the lego block, is observed for both the cubic objects and the cylindrical objects. The spherical objects are kept stable in-hand, with no consistent reduction of position or orientation errors. This behavior results from all the contacts being simulated as contacts between fully rigid objects. This form of contact simulation is particularly relevant for spherical objects where very fine contact management is necessary for repositioning the object.

We attribute the inability of the H-RL policies to minimize the position error to (i) these movements requiring a very explicit reduction of the α_*i*_ for the fingers for which the movement is in the opposite direction to the contact normal and (ii) this explicit coordination being harder to learn when using a model free approach with opposite movements of the object (movements along either the positive or negative direction along the y axis) in the same batch. In addition, exploration where α_*i*_ is substantially skewed toward the upper level policy commands is heavily penalized if the object falls. Despite this, we were able to learn policies where the position errors were minimized by training only with a single position target in the target set. This suggests that a more complex upper level policy is required to learn how to move the object to arbitrary targets.

**Figure 11:**
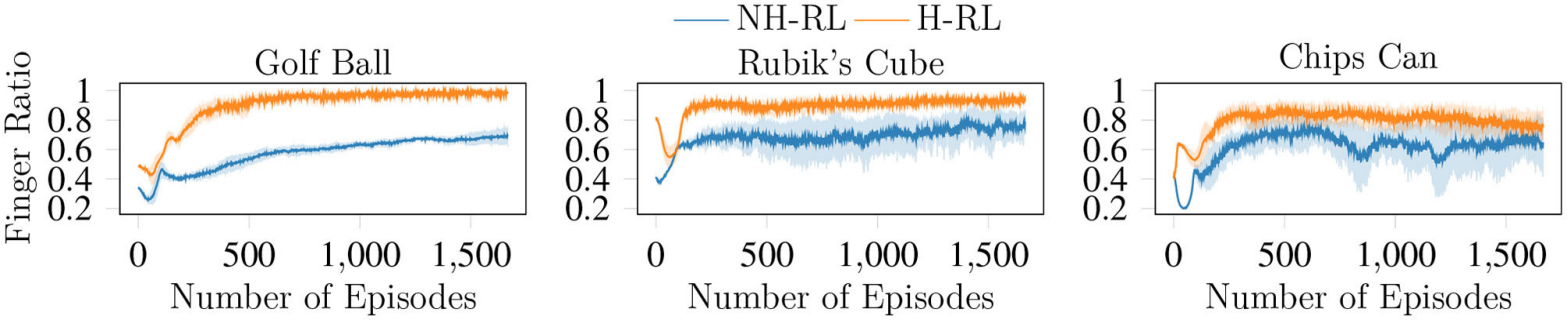
Evolution of the *F* enforcement term with the learning episodes. With *F* converging to one, all fingers that compose the initial grasp are maintained throughout the duration of the movement trajectory.

Finally, we show the effects of the *F* term enforcement, where we wish to keep all fingers of the initial grasps involved in the manipulation action. The evolution of the *F* term with the learning process shown in Figure 11 indicates that the ratio between initial and used fingers increases with the number of episodes, converging to values near the maximum value of one, where all fingers in the initial grasp take part in the manipulation action. Another interesting aspect of the results shown in Figure 11 are the large fluctuations in the *F* ratio in the initial stages of the learning process. While exploring the state action space, the initial policies constantly remove fingers from the object, until a balance is reached between improving the error terms and the *F* ratio simultaneously. This effect is also visible in the cumulative reward curves shown in Figure 7.

### 3.3. Transfer to the Real Robot

**Figure 12:**
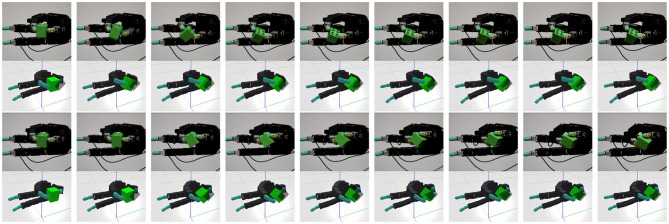
The behavior of two policies trained on the simulator and transferred to the real robot with no additional training. The movements consist of a clockwise (top two rows) or counter clockwise rotation of the object along the axis perpendicular to the palm (bottom two rows).

Several policies were tested on the real robot in order to assess their transfer capabilities. In Figure 12, the movements produced by two policies for the lego block are depicted. These policies were transferred with no further learning on the real robot, displaying similar movements to the ones observed in the simulation environment. While policies correctly transfer to the real robot, the movements are hindered by inaccuracies in the estimated contact normal and by noise on the slip predictors. These estimations are fairly robust for small movements but quickly diverge once contact positions considerably shift. These errors in the contact normal are reduced during the execution of the movement since the α_*i*_ values are providing more control to the upper level policy. Once the upper level policy finishes the desired manipulation movement, and the values of α_*i*_ begging to be shift the control to the low level stabilizers, the contact normal errors become more relevant, often resulting in the object being dropped from the grasp. From the policies tested, the ones that better transferred were the ones for the lego block and the Rubik's cube, which is consistent with the manipulation performances observed in simulation.

## 4. Conclusion

We have proposed a hierarchical decomposition for the in-hand manipulation problem in order to enable learning policies for manipulating unsupported objects. The policies learned in simulation are transferred to a real robot platform where similar manipulation movements are observed. Our decomposition is based on low-level per-digit stabilizing controllers that effectively incorporate tactile feedback to ensure a stable grip during object manipulation and a high-level policy that coordinates digit movement and modulates the influence of the individual low-level controllers. Our decomposition allows for efficient training of high-level policies for dexterous manipulation in simulation on a range of different objects achieving faster learning and higher rewards than its non-hierarchical counterpart. By abstracting and encapsulating tactile feedback in the lower-level controllers, the hierarchical decomposition enables direct transfer of policies that were trained in simulation to a physical system.

An interesting direction for future work is to explore the possibility of learning a single policy that is able to perform all achievable translations and rotations of the grasped object by taking inspiration from recent developments in multi-task reinforcement learning.

## Data Availability Statement

The datasets generated for this study are available on request to the corresponding author.

## Author Contributions

FV executed the work with the assistance of RA with respect to the reinforcement learning approach. JP with respect to the overall architecture of the control approach. All authors contributed to the article and approved the submitted version.

## Conflict of Interest

The authors declare that the research was conducted in the absence of any commercial or financial relationships that could be construed as a potential conflict of interest.
